# A case of synchronous breast and bilateral lung cancers: literature review and considerations for radiation treatment planning

**DOI:** 10.1259/bjrcr.20150464

**Published:** 2015-08-25

**Authors:** Chunzi Jenny Jin, Xiangyang Mei, Conrad B Falkson

**Affiliations:** ^1^Department of Oncology, Cancer Centre of Southeastern Ontario, Kingston General Hospital, Kingston, ON, Canada; ^2^Department of Physics, Cancer Centre of Southeastern Ontario, Kingston General Hospital, Kingston, ON, Canada

## Abstract

Limited literature is available regarding treatment strategies for three concurrent potentially curable malignancies, and only one case of primary breast cancer with bilateral primary lung cancers has been reported. There is no literature available on approaches to radiation treatment planning and delivery in this challenging scenario. We report a case of a 66-year-old female who underwent partial mastectomy and sentinel node biopsy for left-sided breast cancer, pT1cN1(mic). Metastatic work-up revealed bilateral primary lung cancers, biopsy-proven, each Stage cT1N0. Distinguishing synchronous primary tumours from metastatic disease can be challenging. The histological examination suggested three distinct primaries and each was potentially curable. Devising a treatment strategy required balancing the incremental benefits with the toxicity of combining each of the treatments. Stereotactic ablative radiotherapy was the treatment of choice for the patient's lung primaries, as she was deemed a high-risk surgical candidate. Tangential whole breast radiotherapy with regional nodal irradiation was deemed appropriate for her breast cancer. Treatment for all three sites was planned concurrently, taking into account any potential overlap of dose in the composite plan. Each lung lesion was treated with 48 Gy in 4 fractions with stereotactic ablative radiotherapy using volumetric modulated arc therapy technique. The breast and supraclavicular regions were treated with 50 Gy in 25 daily fractions using a field-in-field technique. Optimal clinical outcomes for patients with multiple primary cancers require optimal definitive management. In this unique case of triple primaries, curative-intent radiotherapy to both lungs, the left breast and regional nodes was planned to be given concurrently and treatment was successfully delivered without significant toxicity.

## Background

There is limited literature available regarding treatment strategies for three concurrent potentially curable malignancies, and only one case of primary breast cancer with bilateral primary lung cancers has been reported.^1^ There is no literature available on approaches to radiation treatment planning and delivery in this challenging scenario. We report an extremely rare case of synchronous triple primary cancer of breast and bilateral lungs, all treated with curative-intent radiotherapy.

## Case presentation

A 66-year-old female was found to have an abnormality in the lower outer quadrant of her left breast on screening mammogram. Core biopsy showed infiltrating ductal cancer. Her medical history was significant for smoking, Type 2 diabetes and transient ischaemic attacks.

**Figure 1.**
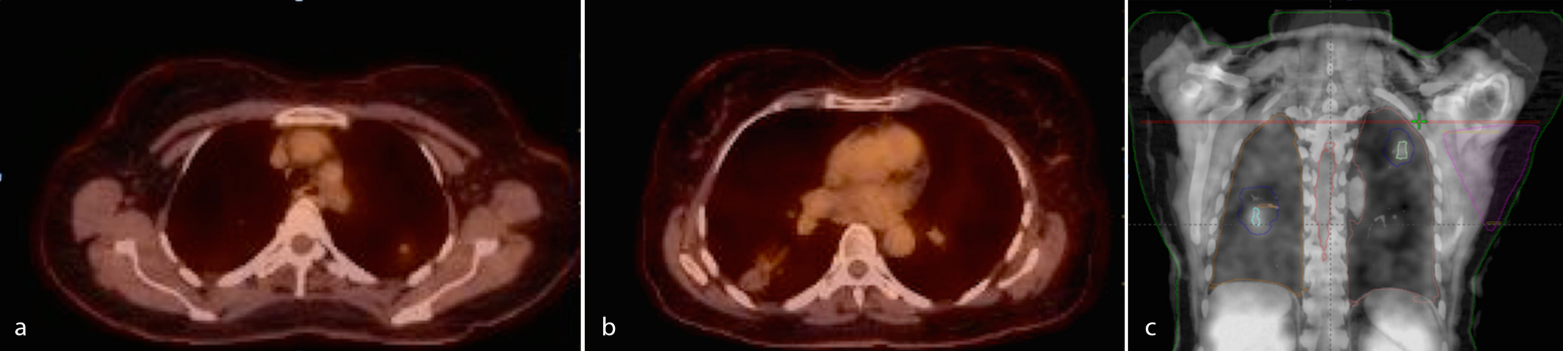
Positron emission tomography-CT scan axial (a, b) and coronal (c) images show fludeoxyglucose-avid lesions in the left upper lobe (maximum standardized uptake value of 1.6) and right lower lobe (maximum standardized uptake value of 2.0).

She underwent left partial mastectomy and sentinel node biopsy. Pathological examination revealed moderately differentiated infiltrating ductal cancer, pT1c (16 mm), N1mi(sn) (0.5 mm); oestrogen and progesterone receptor positive, human epidermal growth factor receptor 2 negative; and Oncotype DX score 21. Post-surgical staging CT scan (node-positive disease) revealed bilateral lung masses: left upper lobe measuring 1.2 cm and right lower lobe measuring 1.8 cm, and both were proven to be positron emission tomography (PET) fludeoxyglucose avid (Figure 1). An MRI of the brain and a bone scan showed no evidence of metastases.

Differential diagnosis included three synchronous primary cancers or metastatic disease. Transthoracic core biopsy of the lung lesions revealed adenocarcinoma acinar type, positive for thyroid transcription factor 1 and Napsin; negative for oestrogen receptor, anaplastic lymphoma kinase, and epidermal growth factor receptor.

The case was discussed at the multidisciplinary tumour board. Histology suggested three distinct curable primaries: T1N1 breast cancer and bilateral T1N0 lung cancers. Management of the breast cancer included partial mastectomy with sentinel node sampling; adjuvant tangential whole breast irradiation (WBI) with regional nodal irradiation (RNI); and an aromatase inhibitor (letrozole). The bilateral lung primaries were planned to be treated with stereotactic ablative radiotherapy (SABR).

The patient underwent four-dimensional CT simulation, with immobilization by abdominal compression for the SABR plans, and free-breathing CT simulation for the breast and regional nodal plans. CT images were fused with those from the PET scan. Gross tumour volumes, clinical target volumes, bilateral lung internal target volumes, planning target volumes, organs at risk and axillary nodal regions were defined as per local policy.

The left and right lung tumours were each treated with 48 Gy in 4 fractions prescribed to the isocentre of each lung lesion, using SABR with volumetric modulated arc therapy technique. A pair of clockwise and counterclockwise partial arcs was utilized, with the collimator set at 30 and 330 degrees to avoid the contralateral lung. A 6 MV photon beam was used.

The breast and regional nodes (supraclavicular and axilla levels I, II and III) were treated to 50 Gy in 25 fractions using a field-in-field technique in a four-field beam arrangement. The breast fields were delineated so that the target (whole breast) was covered by 95–107% of the prescribed dose. Fluence maps were extended 2 cm outside the breast skin to accommodate the respiratory motion of the chest. 6 and 15 MV photon beams were used to achieve adequate dose coverage and dose uniformity for large separation.

**Figure 2.**
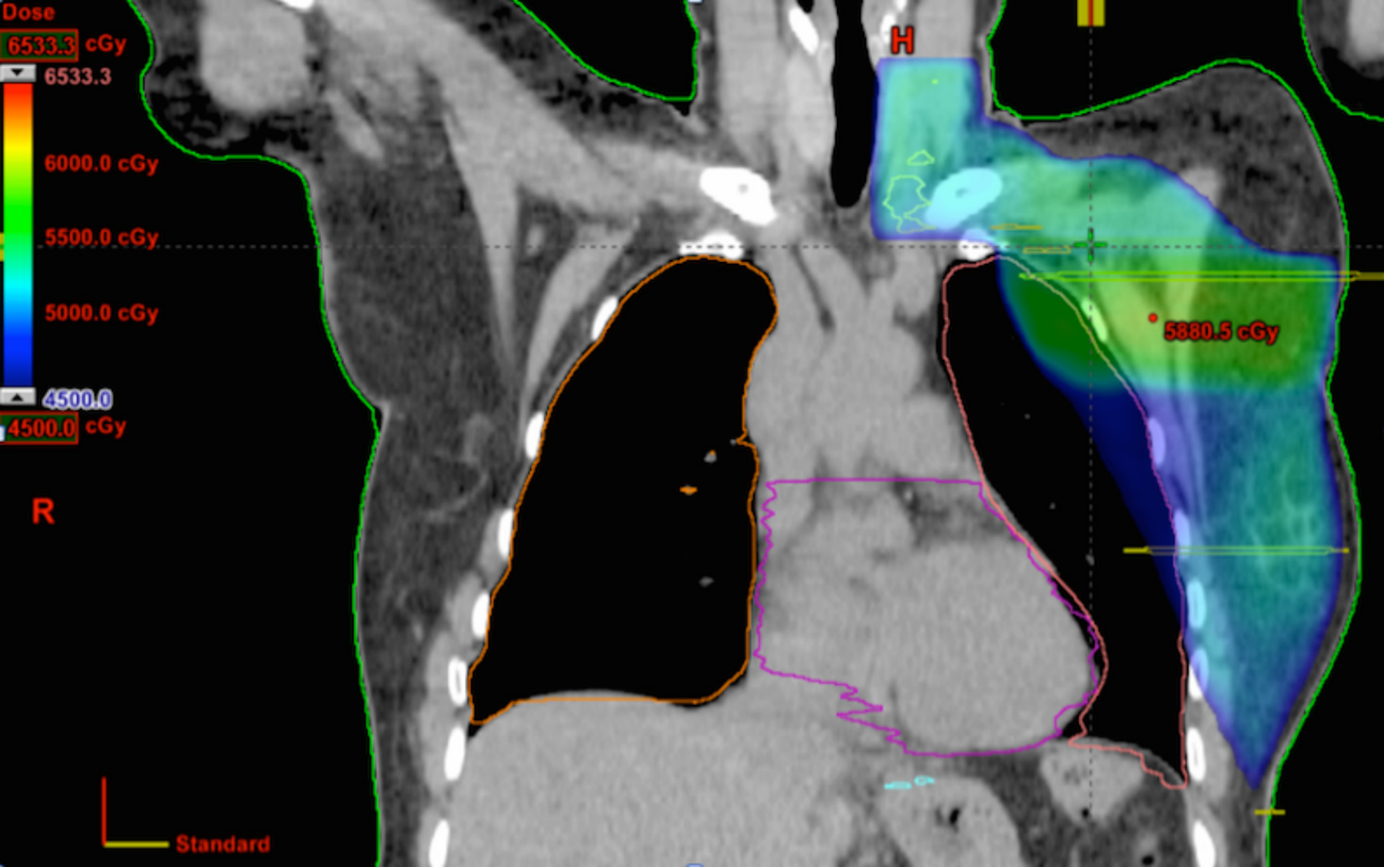
Composite plan showing the region of increased dose in the upper portion of the left breast from the stereotactic ablative radiotherapy plan.

It was planned to treat all three sites concurrently. In the composite plan, all doses and dose coverage were within tolerance limits, despite a region of increased dose in the upper left breast, owing to the contribution from the SABR plan (Figure 2). Tables 1 and 2 show the dosimetric parameters for individual plans and Table 3 shows the equivalent dose in 2 Gy fractions for individual plans and the composite plan. Three safe and deliverable treatment plans were developed.

**Table 1.** Dose parameters of the left breast and regional nodal irradiation plan

Volume of interest	Parameter	Value
Target dose coverage		
Left breast	Volume (cc)	991.01
	D_mean_ (Gy)	49.99
	D_95%_ (Gy)	47.41
	D_99%_ (Gy)	46.03
	D_max_ (Gy)	53.25
	V_52.5Gy_ (%)	0.37
Regional nodes		
Axilla—level I	Volume (cc)	43.75
	D_mean_ (Gy)	48.48
	D_95%_ (Gy)	46.97
	D_99%_ (Gy)	46.52
	D_max_ (Gy)	51.2
	V_52.5Gy_ (%)	0
Axilla—level II	Volume (cc)	15.32
	D_mean_ (Gy)	48.47
	D_95%_ (Gy)	46.00
	D_99%_ (Gy)	45.39
	D_max_ (Gy)	52.81
	V_52.5Gy_ (%)	0.04
Axilla—level III	Volume (cc)	11.45
	D_mean_ (Gy)	51.2
	D_95%_ (Gy)	48.6
	D_99%_ (Gy)	47.9
	D_max_ (Gy)	53.86
	V_52.5Gy_ (%)	15.90
Supraclavicular	Volume (cc)	29.5
	D_mean_ (Gy)	50.71
	D_95%_ (Gy)	48.00
	D_99%_ (Gy)	44.38
	D_max_ (Gy)	53.2
	V_52.5Gy_ (%)	2.35
Internal mammary	Volume (cc)	6.4
	D_mean_ (Gy)	7.71
Organs at risk		
Heart	D_mean_ (Gy)	1.55
	V_5Gy_ (%)	3.06
	V_10Gy_ (%)	0.60
	V_20Gy_ (%)	0
Left anterior descending coronary artery	D_mean_ (Gy)	4.27
Left lung	D_mean_ (Gy)	12.93
	V_5Gy_ (%)	50.70
	V_10Gy_ (%)	33.23
	V_20Gy_ (%)	23.21
	V_30Gy_ (%)	19.88
	V_40Gy_ (%)	15.50
Right lung	D_mean_ (Gy)	0.185
	V_5Gy_ (%)	0
Contralateral right breast	D_mean_ (Gy)	0.11

**Table 2.** Dose parameters of bilateral lung stereotactic ablative radiotherapy plans

Volume of interest	Parameter	Right lung	Left lung	Plan sum—right and left lungs
Target dose coverage				
ITV	Volume (cc)	16.76	4.24	—
	D_95%_ (Gy)	52.77	53.35	—
	D_99%_ (Gy)	51.71	52.63	—
	V_50.4Gy_ (%)	99.99	100.00	—
	V_52.8Gy_ (%)	94.82	97.88	—
PTV	Volume (cc)	46.71	17.00	—
	D_95%_ (Gy)	48.00	48.03	—
	D_99%_ (Gy)	46.14	46.40	—
	V_50.4Gy_ (%)	80.96	81.37	—
	V_52.8Gy_ (%)	54.80	56.20	—
Organs at risk				
Contralateral lung	D_mean_ (Gy)	1.37	0.60	—
Total lung	D_mean_ (Gy)	5.85	2.34	8.26
	V_20Gy_ (%)	8.77	2.62	11.73
Lung—basic function	V_11.6Gy_ (cc)	410	165	595
Lung—pneumonitis	V_12.4Gy_ (cc)	388	150	553
Aorta	D_max_ (Gy)	8.98	19.55	19.98
Pulmonary artery	D_max_ (Gy))	10.85	5.74	11.10
Spinal canal	D_max_ (Gy)	16.60	6.93	16.84
Heart	D_max_ (Gy)	11.54	0.39	11.70
	D_mean_ (Gy)	2.16	0.10	2.26
Left anterior descending coronary artery	D_max_ (Gy)	4.08	0.28	1.09
Oesophagus	D_max_ (Gy)	9.67	9.27	9.90
Chest wall (rib)	D_max_ (Gy)	53.5	56.7	56.46
	V_32Gy_ (cc)	5.36	11.87	—
	V_30Gy_ (cc)	8.80	14.70	—
Proximal trachea	D_max_ (Gy)	0.67	6.35	6.71
Proximal bronchial tree	D_max_ (Gy)	15.45	5.32	15.67
Right breast	D_mean_ (Gy)	4.73	0.41	5.14
Left breast	D_mean_ (Gy)	0.91	1.72	2.63

ITV, internal target volume; PTV, planned target volume.

**Table 3.** EQD2 for individual plans and plan sum

	Left breast and regional nodes 50 Gy/25		Right lung 48 Gy/4		Left lung 48 Gy/4		Cumulative plan sum
	D_mean_ (Gy)	EQD2 †⧫ (Gy)	D_mean_ (Gy)	EQD2 †⧫ (Gy)	D_mean_ (Gy)	EQD2 †⧫ (Gy)	EQD2 (Gy)
Left breast	49.99	49.99	0.91	0.67	1.72	1.31	51.97
Regional nodes							
Axilla—level I	48.48	48.48	0.53	0.38	5.73	5.24	54.10
Axilla—level II	48.47	48.47	0.13	0.09	5.86	5.23	53.79
Axilla—level III	51.20	51.20	0.15	0.11	0.73	0.53	51.84
Internal mammary	7.71	7.71	0.40	0.28	8.59	8.78	16.77
Supraclavicular	50.71	50.71	0.11	0.08	0.36	0.26	51.05
Right lung ITV	0.16	0.16	55.82	111.43	0.15	0.13	111.72
Right lung PTV	0.16	0.16	53.09	102.96	0.16	0.13	103.25
Left lung ITV	4.44	4.44	0.29	0.24	55.26	109.67	114.35
Left lung PTV	4.70	4.70	0.30	0.25	52.85	102.23	107.18
Organs at risk							
Left lung*	13.16	13.16	1.38	0.92	4.47	3.68	17.76
Right lung*	0.19	0.19	9.08	9.57	0.6	0.38	10.14
Total lung*	6.01	6.01	5.62	4.95	2.34	1.68	12.64
Heart	1.55	1.55	2.16	1.53	0.10	0.06	3.14
Left anterior descending coronary artery	4.27	4.27	0.99	0.64	0.09	0.05	4.96
Contralateral right breast	0.11	0.11	4.73	3.96	0.41	0.25	4.32

EQD2 = Equivalent dose in 2 Gy per fraction; ITV, internal target volume; PTV, planned target volume.

*Organ at risk lung volumes do not include ITV;

†α/β = 10 (tumour control);

⧫ α/β = 3 (late normal tissue);

The left and right lung were concurrently treated with 48 Gy in 4 fractions, delivered with abdominal compression, treating each side on alternate days, for a total 8-day course. Patient position was verified with daily cone-beam CT pre- and post-treatment. Each daily lung treatment, including patient setup and beam delivery, was completed in a 15-minute slot with intensity-modulated radiotherapy.

Following completion of lung treatments, the breast and regional nodes were treated to 50 Gy in 25 fractions, delivered 5 days per week, without breathing control. Field position was verified using kV–kV imaging.

The patient completed all her treatments without toxicity. She developed dyspnoea and cough 5 weeks post lung radiotherapy and within 1 week of completing breast radiotherapy. She did not feel her symptoms warranted therapy, and given her diabetic history, steroids were withheld. Her CT scan was consistent with post-radiotherapy changes. Pulmonary function tests showed mild restrictive defect, forced expiratory volume in 1 s/forced vital capacity = 56% (compared with 66% pre-radiotherapy). Repeat CT scan 6 months post radiotherapy showed stable lung findings. Examination of the breast skin revealed excellent cosmetic results.

## Discussion

This case presents an approach to diagnosis and management, with a description of radiation treatment planning and delivery, in an extremely rare setting of synchronous triple primary cancers of the left breast and bilateral lungs, all treated with curative intent. There are a limited number of reports on three synchronous carcinomas, and only one case of breast cancer with bilateral primary lung cancers has been previously described in the literature.^1^

Synchronous carcinomas are tumours diagnosed within 6 months of the index cancer. Synchronous primary lung cancers have been reported to occur in approximately 0.5% of lung cancer patients and are more frequently located in the same lung.^2^ The two lung primaries must either differ in histology or be located in different segments, without evidence of extrapulmonary or lymphatic spread.^3^ While the aetiology of multifocal primary lung cancers may be linked to field cancerization, there are no known predisposing factors common to both lung and breast malignancies. Synchronous lung and breast cancers are rare, affecting fewer than 0.5% of breast cancer patients.^1^ Pulmonary nodules arising in females with breast cancer have been reported to reflect primary lung cancer in 55%, metastatic disease in 37% and a benign lesion in 8% of cases.^1^ Thus, pathological evaluation of an isolated pulmonary lesion is indicated in females with breast cancer, as half of the cases could potentially receive curative treatment.

Distinguishing synchronous primary tumours from metastatic disease can be challenging. Histology, immunohistochemistry, PET-CT scan and multidisciplinary conference discussion are merited. Our philosophy, in case of doubt, is to treat each tumour as a separate primary and treat with curative intent. Determining a treatment strategy requires balancing the incremental benefits with toxicity of combining treatments.

SABR was the treatment of choice for our patient's lung primaries, as she was deemed a high-risk surgical candidate. SABR offers outcomes similar to those of surgical resection, with lower risk of treatment-related morbidity in Stage I non-small cell lung cancer.^4^ Compared with conventional radiotherapy, SABR offers superior outcomes.^5^ For early-stage multiple primary lung cancers, there is limited evidence suggesting comparable outcomes with SABR and surgery.^6^

Tangential WBI with RNI was deemed appropriate for our patient's breast cancer. Surgical options were also considered to avoid potential toxicity associated with irradiation of all sites. However, in the setting of node-positive disease, mastectomy would not have spared our patient of treatment with adjuvant WBI. WBI confers local control and survival benefit following breast-conserving surgery for early-stage breast cancer and following mastectomy for node-positive disease.^7–9^ Furthermore, RNI offers comparable local control and survival advantages to patients undergoing axillary node dissection, with only modest toxicity.^9^ Axillary and supraclavicular irradiation is most commonly recommended.^9^ There is insufficient data to recommend routine radiotherapy to the internal mammary nodes. Although internal mammary irradiation is often delivered as part of nodal irradiation, data on the benefit of internal mammary radiotherapy specifically are limited and contradictory.^9^ We specifically chose not to cover internal mammary nodes so as to limit excess dose to the heart and lungs.

Breathing control was not applied during the simulation and treatment of the breast and regional nodes; thus, the planned dose may not have been equal to the actual delivered dose. Motion management techniques include deep inspiration breath-hold (DIBH), forced shallow breathing with abdominal compression, respiratory gating and tracking. In the treatment of our patient's bilateral lung cancers, abdominal compression was beneficial given the significant tumour motion in the craniocaudal direction. In the treatment of her breast cancer, there were no concerns with geographical miss of the target volume with intrafraction motion induced by breathing: chest wall motion is relatively limited during quiet breathing; large margins are created by extending the fluence map 2 cm beyond the breast skin; and there is a gradual change of beam intensity in the motion direction. Rather, the aim of motion management in WBI is to reduce radiation doses to normal structures. DIBH can be an effective method of reducing radiation dose to the heart to decrease the risk of cardiac toxicity, particularly for patients undergoing left-sided breast radiotherapy, and may confer an additional benefit in reducing the density of healthy lung tissue in the radiation field. DIBH has subsequently been implemented in the treatment of left-sided breast cancers at our centre.

Concurrent treatment planning of all sites of multiple primary cancers occurring within proximate body regions avoids change in patient positioning and allows for the evaluation of a composite plan to identify any potential dose overlap. In this case, three clinically acceptable treatment plans were devised and we do not anticipate excess long-term toxicity.

The availability of literature on prognosis of patients with multiple primary cancers is limited. Breast cancer patients with synchronous lung cancers appear to fare worse owing to increased mortality from lung cancer.^3,10^ While synchronous lung nodules in different lobes are staged as metastatic disease (Stage IV), survival rates following definitive treatment are still comparable to those with IB and IIA lesions.^3^

## Conclusions

Optimal clinical outcomes for patients with multiple primary cancers require optimal definitive management. In this unique case of triple primaries, curative-intent radiotherapy to both lungs, the left breast and regional nodes was planned concurrently and treatment was successfully delivered without significant toxicity.

## Learning points

1. To distinguish synchronous primaries from metastases, histology, immunohistochemistry, PET-CT scan and multidisciplinary tumour board discussion are merited. When in doubt, treat as separate, curable tumours if feasible.
2. Devising a treatment strategy requires balancing the incremental benefits with the toxicity of combining each treatment.
3. Simultaneous planning of all sites is needed to devise a composite plan by taking into account any potential dose overlap.
4. Respiratory motion control is increasingly being used in the irradiation of tumour sites affected by respiratory motion, including lung and breast. This should be considered in order to obtain the precise dose for the volume of interest and to reduce risk of normal tissue complications when larger treatment volumes are required for several tumour sites.

## Consent

Informed consent was obtained from the patient for publication of this case report, including accompanying images.
